# Supplementary material for: Demonstration of Tunable Control over a Delayed-Release Vaccine Using Atomic Layer Deposition
Source: Vaccines (Basel). 2024 Jul 11;12(7):761. doi: 10.3390/vaccines12070761 (PMC11281649; doi:10.3390/vaccines12070761)
Supplement: Supplementary file 1 [file vaccines-12-00761-s001.zip › vaccines-3063980-supplementary.pdf]

vaccines

MDPI

Supplementary Table S1

The 4PL terms generated from in vitro dissolution of powder coated with 100 ALD cycles and the 95% confidence intervals. The parameters include the initial percent release (A), the final percent release (D), the time (hours) to inflection point of the curve (C), and the slope at the inflection point (B).

4PL Term	100 cycles ALD *(95% CI)*
**A – the initial % release**	**4.496** *(-10.30 to 18.22)*
**D – the final % release**	**99.69** *(94.11 to 105.4)*
**C – the time to inflection point**	**15.93** *(14.08 to17.72)*
**B – the slope at inflection point**	**8.201** *(undetermined)*

Supplementary Table S2

The 4PL terms generated from in vitro dissolution of powder coated with 50, 250, 500, and 1000 ALD cycles and the 95% confidence intervals. The parameters include the initial percent release (A), the final percent release (D), the time (hours) to inflection point of the curve (C), and the slope at the inflection point (B).

4PL Term	50 cycles *(95% CI)*	250 cycles *(95% CI)*	500 cycles *(95% CI)*	1000 cycles *(95% CI)*
**A – the initial % release**	**8.089** *(5.613 to 10.54)*	**2.660** *(-0.4875 to 5.758)*	**3.171** *(0.4373 to 5.873)*	**-0.5687** *(-6.353 to 5.168)*
**D – the final % release**	**98.74** *(92.39 to106.4)*	**92.76** *(90.93 to 94.61)*	**100** *(96.81 to 103.4)*	**99.82** *(92.93 to 106.9)*
**C – the time to inflection point**	**9.401** *(9.262 to 9.537)*	**25.94** *(25.25 to 26.63)*	**53.47** *(52.39 to 54.57)*	**136.5** *(132.5 to 140.8)*
**B – the slope at inflection point**	**26.57** *(undetermined)*	**10.96** *(8.730 to 14.15)*	**12.44** *(undetermined)*	**19.88** *(13.66 to 31.79)*
